# Investigating Macular Tissue Integrity Index as a Novel Biomarker in Geographic Atrophy

**DOI:** 10.1016/j.xops.2025.100871

**Published:** 2025-06-30

**Authors:** Bethany M. Erb, Elaine Botros, Thomas F. Saunders, Anna-Maria Haas, Rick Voland, Rachel Linderman, Amitha Domalpally, Karl G. Csaky

**Affiliations:** 1 Wisconsin Reading Center, Department of Ophthalmology and Visual Sciences, University of Wisconsin-Madison, Madison, Wisconsin; 2 Karl Landsteiner Institute for Retinal Research and Imaging, Vienna, Austria; 3 Retina Foundation of the Southwest, Dallas, Texas

**Keywords:** Age-related macular degeneration, Autofluorescence, Ellipsoid zone, Geographic atrophy

## Abstract

**Purpose:**

The rate of geographic atrophy (GA) enlargement is commonly used as an outcome in clinical trials. However, this metric typically lacks specificity for central macular involvement and structure–function relationships. We propose a targeted approach in monitoring GA progression within the central macula, highlighting the limited benefit of including GA expansion beyond the 3-mm perifoveal zone when analyzing visual function. This study evaluates how retinal tissue and photoreceptor integrity within the central 1-mm and 3-mm circles centered on the fovea correlate with visual function.

**Design:**

Retrospective, longitudinal analysis of a GA clinical trial cohort.

**Subjects:**

Forty-three eyes from 43 participants enrolled in GA clinical trials.

**Methods:**

Baseline and 1-year fundus autofluorescence (FAF) and OCT scans were analyzed. The percentages of non-GA areas within the 1-mm and 3-mm circles centered on the fovea were quantified to calculate the Macular Tissue Integrity Index (MTII) using FAF images. The percentages of intact ellipsoid zones within the same circles were used to define the EZ Integrity Index (EZII). Longitudinal changes in MTII and EZII were compared to overall GA area growth and change in visual acuity (VA).

**Main Outcome Measures:**

Correlations between MTII, EZII, GA area, and VA (best-corrected VA [BCVA] and low-luminance VA [LLVA]) were assessed.

**Results:**

Macular Tissue Integrity Index and EZII within the central 1 mm correlated significantly with BCVA (R^2^ = 0.20, P = 0.003 and R^2^ = 0.29, P < 0.001, respectively), while EZII in the 3-mm zone correlated with both BCVA and LLVA (R^2^ = 0.17, P < 0.01 for both). Changes in MTII or EZII over time were not associated with GA area growth or with baseline integrity indices.

**Conclusions:**

Macular Tissue Integrity Index and EZII are novel biomarkers for macular photoreceptor integrity, with distinct correlations to BCVA and LLVA depending on the measurement zone. These findings support the utility of MTII and EZII in assessing macular integrity and highlight the heterogeneity of GA progression, warranting further validation in larger studies.

Age-related macular degeneration (AMD) is the leading cause of blindness in the modern world and threatens to affect 288 million people by 2040.^1^ Geographic atrophy (GA) and neovascular AMD represent late stages of the disease which can lead to irreversible vision loss. Geographic atrophy is characterized by the progressive loss of retinal pigment epithelium, photoreceptors, and choriocapillaris.^2^ While treatments exist for neovascular AMD, therapies for GA remain limited.

In 2023, the US Food and Drug Administration approved 2 intravitreal complement inhibitors—pegcetacoplan and avacincaptad pegol—as the first pharmaceutical treatments for GA.^3^^,^^4^ While these drugs reduce GA lesion growth, neither has been shown to provide any significant effect on functional vision outcomes, such as best-corrected visual acuity (BCVA) or reading speed, leading the European Medicines Agency to withhold market authorization.^5^^,^^6^

The measurement of overall GA enlargement on fundus autofluorescence (FAF), the current gold standard in clinical trials, often fails to accurately delineate the critical central macular region and its structure-function relationship.^7^ Therefore, calls have been made to reconsider the use of GA lesion growth as a surrogate end point for visual acuity (VA) in clinical trials.^8^ A primary concern is that GA growth does not necessarily correlate with VA changes, as lesions may expand outwardly rather than progressing toward the macula, which is the region critical for visual function.^9^ As such, drugs that primarily prevent overall GA enlargement, without addressing progression toward the macula, may not preserve central vision. Measuring how much of the macula remains intact (non-GA area) could serve as an important end point in clinical trials.^10^

Emerging research proposes that ellipsoid zone (EZ) integrity, a key indicator of photoreceptor health, may serve as a better end point for assessing GA progression.^11^ Studies have shown that loss of the EZ at GA margins frequently precedes photoreceptor damage, signaling the progression of GA area expansion.^12^^,^^13^ Consequently, the prevention of EZ loss has been accepted as an end point in GA clinical trials.^12^ While EZ loss is a better therapeutic outcome measure than GA area, the issue of structure-function discrepancy and lack of meaningful functional improvement from a patient perspective remains.^14^

To address this gap, metrics that better capture short-term functional benefits and disease activity within or around the macula are needed. Incorporating measures such as the area of non-GA tissue within the central macula on FAF or the extent of intact EZ on OCT could represent novel end points for evaluating functional outcomes. These metrics would better align with the goal of preserving functional vision by targeting macular integrity and preservation. This study proposes Macular Tissue Integrity Index (MTII) and EZ Integrity Index (EZII) as alternative biomarkers, quantifying tissue integrity in the 1-mm and 3-mm areas around the fovea. Macular Tissue Integrity Index uses FAF to monitor non-GA areas in the central macula, whereas EZII uses OCT imaging to evaluate the integrity of the EZ in the corresponding areas. The present study aims to evaluate these novel biomarkers and how they correlate to visual function as measured by BCVA and low-luminance VA (LLVA).

## Methods

### Study Design

This study performed post hoc analysis on 18-month data from a GlaxoSmithKline trial (ClinicalTrials.gov identifier: NCT01342926) for which study design, primary outcomes, and patient demographics have been previously published. The study was a multicenter, randomized, placebo-controlled, parallel-group phase I study investigating the safety, tolerability, efficacy, pharmacokinetics, and pharmacodynamics of GSK933776—a humanized immunoglobulin G monoclonal antibody—in adult patients with GA secondary to AMD.^15^ The study showed no reduction in the rate of GA growth in patients treated with GSK933776 compared with those receiving placebo, nor were differences noted between the groups in any of the measures of visual function assessed (BCVA, LLVA, reading speed). The study was conducted under institutional review board approval (University of Wisconsin-Madison School of Medicine & Public Health) at each site and written informed consent was obtained from all study participants. The research followed the tenets of the Declaration of Helsinki and complied with the Health Insurance Portability and Accountability Act.

A total of 141 participants were enrolled and completed study follow-up. Of these, 104 participants were included in the treatment groups and 37 in the control group. All participants were required to have well-demarcated GA resulting from AMD with a total area of 1.9 to 17 mm^2^ (approximately 0.75–6.7 disc areas) measured on color fundus photographs in the study eye per original study protocol. For multifocal GA, ≥1 of the foci had to have an area of ≥1.9 mm^2^ (0.75 disc areas) and the total area of GA had to measure ≤17 mm^2^ (6.7 disc areas). For the purposes of this subanalysis, only participants with paired Heidelberg FAF and OCT images of adequate quality were included, reducing the study population to 63. To ensure meaningful structural measurements, participants with minimal preserved tissue in the central 1 mm on FAF were excluded. A minimum of 0.05 mm^2^ was adopted to mirror the minimum size criteria needed for the diagnosis of GA as outlined by the Classification of Atrophy Meeting group.^16^ This left 43 participants for FAF analysis. OCT images from an additional participant were removed due to file corruption, leaving a total of 42 participants for OCT analysis.

### Imaging and Grading Protocol

**Figure 1:**
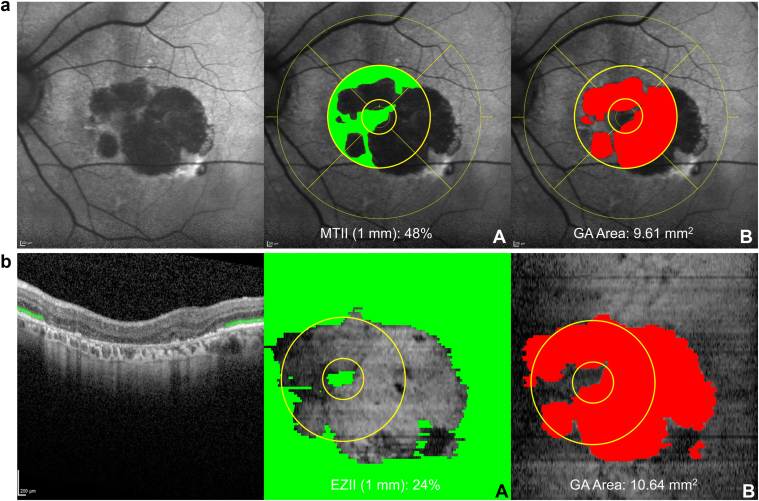
**A,** Fundus autofluorescence images of a participant with GA demonstrating annotation of intact retinal tissue (**a**) and GA (**b**). **B,** OCT images of a participant with GA demonstrating annotation of intact ellipsoid zone (**a**) and GA (**b**). EZII = Ellipsoid Zone Integrity Index; GA = geographic atrophy; MTII = Macular Tissue Integrity Index.

Fundus autofluorescence images were captured using Heidelberg Spectralis (Heidelberg Engineering). Macular Tissue Integrity Index assessment of these images was performed by 2 trained graders at the Wisconsin Reading Center within the Heidelberg Eye Explorer software. Using manual planimetry, graders segmented the borders of all GA lesions. Geographic atrophy was defined as an area or areas of well-defined hypoautofluorescence. The minimum size criteria were 250 microns in the smallest diameter or area ≥0.05 mm^2^. Foveal involvement was confirmed using Heidelberg Spectralis OCT images. Graders evaluated individual B-scans to determine whether outer retinal atrophy extended through the foveal center and correlated this with FAF images. Geographic atrophy area was calculated for the entire image. The area of intact tissue within the 1-mm and 3-mm circles around the fovea was calculated by subtracting GA area within from the known area of these circles as shown in Figure 1A. As images were annotated within the Heidelberg Eye Explorer software using the built-in ETDRS grid, the actual diameter of the 1-mm and 2-mm circles was 1.2 mm and 3.6 mm, respectively (1.2 mm circle = 1.13 mm^2^ area and 3.6 mm circle = 10.18 mm^2^ area). This area was then used to calculate the MTII, that is, the percentage of non-GA within the respective circles.

Ellipsoid Zone Integrity Index assessment was performed by 2 trained graders (BME, EB) at the Wisconsin Reading Center using Heidelberg Spectralis OCT images. Using 3D Slicer (v5.6.2) (http://www.slicer.org), a free and open-source software package for image analysis, graders manually annotated EZ integrity using an edge detection approach. Ellipsoid zone integrity was defined as the continuous presence of a second hyperreflective outer retinal band without any disruption of the OCT signal. Annotating from the left lateral edge of intact EZ, graders continued to the point of noticeable EZ loss, before pausing and then resuming at the next identifiable area of intact EZ. This start-stop pattern was followed until the lateral right edge of the B-scan was reached. This process was repeated for each B-scan until preserved EZ was fully mapped in the corresponding en face image (Fig 1B). Any presence of EZ, independent of thickness, was considered as intact EZ. Ellipsoid zone was only considered absent when no portion of the layer was visible. Graders were trained to use both the en face and B-scan views using 3D Slicer, actively cross-referencing both perspectives to confirm EZ continuity or disruption. The en face view was used to verify the spatial continuity of EZ integrity and to help the grader detect potential gaps or breaks in the annotations, prompting reassessment and verification, thus ensuring greater accuracy in capturing EZ integrity. The en face segmentation map produced through this process was then used to calculate intact EZ area for the entire image as well as within the 1-mm and 3-mm circles around the fovea as shown in Figure 1B. In the case of OCT measurements, the ETDRS grid used had central circles of 1 mm and 3 mm in diameter (1 mm circle = 0.79 mm^2^ area and 3 mm circle = 7.07 mm^2^ area). These area measurements were used to calculate EZII, that is, the percentage of intact EZ within the respective circles.

Both FAF and OCT imaging were further used to characterize GA focality (unifocal or multifocal) and foveal involvement (subfoveal or foveal sparing). Additionally, graders were asked to evaluate images for the presence of reticular pseudodrusen and to classify the pattern in the junctional zone of GA. A senior grader (TFS) evaluated and adjudicated image sets from each grader for each modality, to create a single set of images to be used in analysis.

### Statistical Analysis

Univariant regressions were used to evaluate the relationship of MTII, EZII, and GA area with BCVA and LLVA. Scatterplots were generated to visualize the relationship between BCVA (logarithm of the minimum angle of resolution) and MTII, EZII, and GA area. The categorical variables were summarized as n (%) and continuous variables were summarized as mean ± standard deviation (SD). Statistical significance was set at *P* < 0.05. All analyses were conducted using R software (v4.2.1, R Foundation for Statistical Computing).

## Results

A total of 43 eyes from 43 participants were included in the study. Baseline demographics and imaging features are summarized in Table 1, Table 2. Geographic atrophy was multifocal in 74% of cases, with 42% of lesions located subfoveally. Reticular pseudodrusen were present in 16 eyes (37%), and the junctional zone pattern was predominantly banded or diffuse, being present in 38 eyes (88%). The mean baseline GA area was 8.87 (SD 5.34) mm^2^ with FAF and 9.4 (SD 2.68) mm^2^ with OCT. The MTII percentage with FAF in 1 and 3 mm was 51% and 48% and EZII with OCT was 42% and 38%, respectively. The mean baseline BCVA was 67.9 ± 15 ETDRS letters, and the mean LLVA was 43 ± 19.5 ETDRS letters.

**Table 1:** Participant Demographics

Characteristic	n	%
Gender		
Male	16	37
Female	27	63
Ethnicity		
Not Hispanic or Latino	43	100
Race		
White or Caucasian	43	100
Smoking status		
Never smoked	16	37
Former smoker	27	63
Genetics		
SNP rs1744077		
Carrier (GG/AG)	23	53
Noncarrier (AA)	16	37
SNP rs4698775		
Carrier (GG/GT)	22	51
Noncarrier (TT)	17	40

SNP = single nucleotide polymorphism.

**Table 2:** Baseline Fundus Autofluorescence Features

Feature	n	%
GA focality		
Unifocal	11	26
Multifocal	32	74
GA location		
Subfoveal	18	42
Foveal sparing	25	58
RPD		
Present	16	37
Absent	27	63
Junctional zone pattern		
Banded or diffuse	38	88
Focal or patchy	5	12

GA = geographic atrophy; RPD = reticular pseudodrusen.

### Baseline Structure–Function Correlations

**Figure 2:**
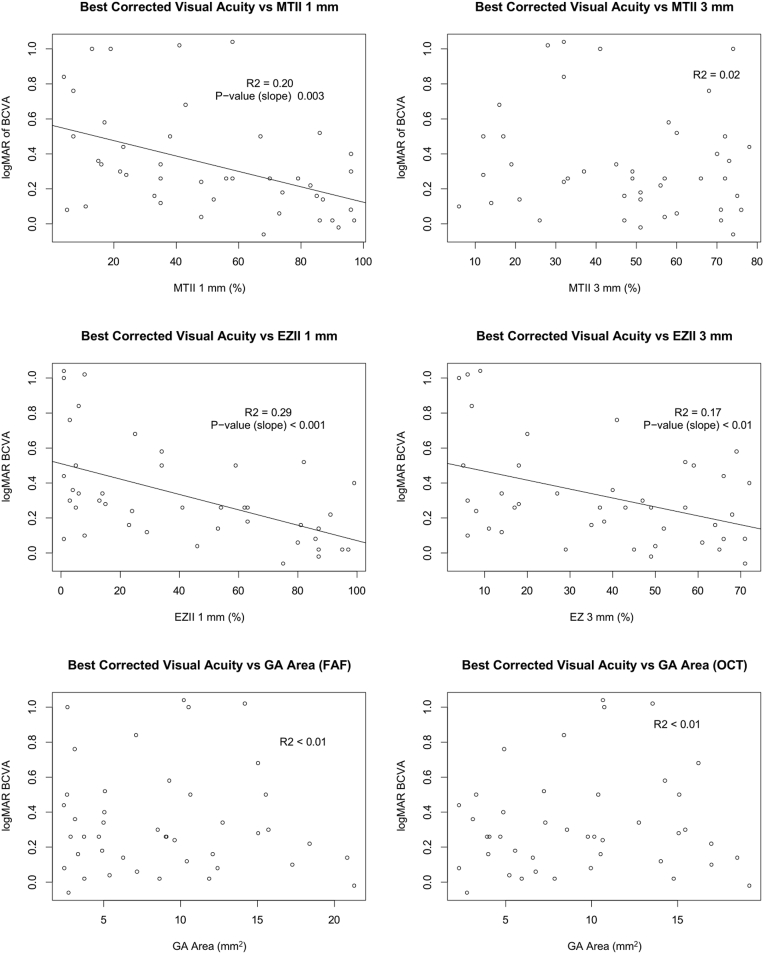
Baseline analysis of MTII (top), EZII (middle), and GA area (bottom) vs. BCVA. BCVA = best-corrected visual acuity; EZII = Ellipsoid Zone Integrity Index; FAF = fundus autofluorescence; GA = geographic atrophy; logMAR = logarithm of the minimum angle of resolution; MTII = Macular Tissue Integrity Index; OCT = Ocular Computed Tomography.

Figure 2 and Figure S1 (available at www.ophthalmologyscience.org) illustrate the relationship of baseline MTII, EZII, and GA area with BCVA and LLVA. Within the central 1-mm circle, both MTII and EZII were significantly correlated with BCVA (R^2^ = 0.20, *P* = 0.003 and R^2^ = 0.29, *P* < 0.001); however, neither MTII nor EZII showed significant correlation with LLVA (R^2^ = 0.03 and R^2^ = 0.02, respectively). In the 3-mm circle, MTII showed no significant association with BCVA (R^2^ = 0.02) or LLVA (R^2^ = 0.05), while EZII maintained a significant correlation with both BCVA (R^2^ = 0.17, *P* < 0.01) and LLVA (R^2^ = 0.17, *P* < 0.01). Geographic atrophy area, measured using both FAF and OCT, showed no significant correlation with either BCVA (R^2^ < 0.01 for both) or LLVA (R^2^ < 0.01 and R^2^ = 0.06, respectively). These findings suggest that both MTII and EZII within the central 1 mm are associated with BCVA, while EZII in the 3-mm circle is associated with both BCVA and LLVA.

**Figure 3:**
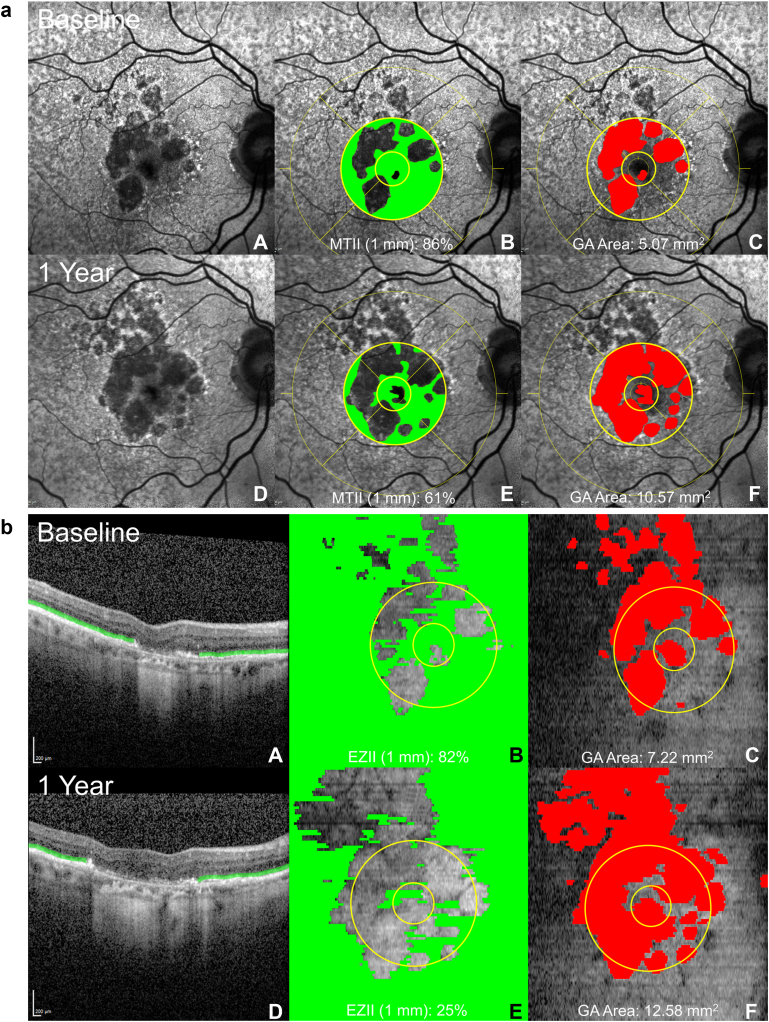
**a,** Fundus autofluorescence images of a participant with GA demonstrating change in MTII and GA between baseline and 1-year follow-up. **b,** OCT images of a participant with GA demonstrating change in EZII and GA area between baseline and 1-year follow-up. **A** and **D** show the raw image at baseline and year 1, **B** and **E** show MTII/ EZII, **C** and **F** show GA area. EZII = Ellipsoid Zone Integrity Index; GA = geographic atrophy; MTII = Macular Tissue Integrity Index.

Table 3 summarizes the baseline, 1 year, and change values for MTII, EZII, and GA area. The annual growth rate for GA area was comparable between FAF and OCT, with a mean increase of 1.90 ± 1.39 mm^2^ and 1.62 ± 1.32 mm^2^, respectively. Similarly, the changes in MTII and EZII were consistent across modalities, with both indices showing a decline of approximately 8% to 10% per year within the central 1-mm and 3-mm circles. These findings highlight a similar rate of structural decline observed with both imaging methods. Figure 3A, B illustrate the changes in MTII and EZII over a 1-year period.

**Table 3:** Baseline, 1 Yr, and Change Values for MTII, EZII, Intact and GA Area (FAF), and Intact EZ and GA Area (OCT)

	Baseline		1 Year		Change	
	Mean	SD	Mean	SD	Mean	SD
FAF (n = 43)						
MTII 1 mm (%)	51	31	40	31	11	8
MTII 3 mm (%)	48	22	40	22	8	5
Intact area 1 mm (mm2)	0.57	0.35	0.45	0.35	0.12	0.09
Intact area 3 mm (mm2)	4.86	2.20	4.03	2.22	0.83	0.49
GA area (mm2)	8.87	5.34	10.77	5.98	1.90	1.39
OCT (n = 42)						
EZII 1 mm (%)	42	35	34	33	8	12
EZII 3 mm (%)	38	23	30	22	9	8
Intact EZ area 1 mm (mm2)	0.33	0.27	0.27	0.26	0.06	0.09
Intact EZ area 3 mm (mm2)	2.68	1.65	2.08	1.52	0.60	0.57
GA area (mm2)	9.40	4.93	11.02	5.45	1.62	1.32

EZ = ellipsoid zone; EZII = Ellipsoid Zone Integrity Index; FAF = fundus autofluorescence; GA = geographic atrophy; MTII = Macular Tissue Integrity Index; SD = standard deviation.

### Change in MTII/EZII over 12 Months

**Figure 4:**
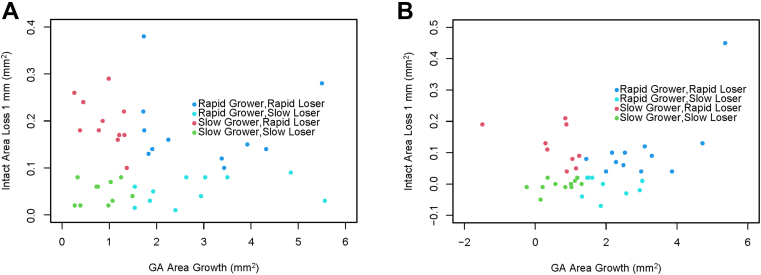
**A,** Loss of intact area compared to GA growth on FAF over 1-year follow-up. **B,** Loss of intact ellipsoid zone area compared to GA area growth on OCT over 1-year follow-up. FAF = fundus autofluorescence; GA = geographic atrophy.

Figure 4A compares the loss of intact area within the central 1 mm to the growth of GA area over 1 year in FAF images, whereas Figure 4B compares the loss of intact EZ area with GA area enlargement on OCT over the same time period. No significant correlation was observed between the enlargement of GA and decrease in MTII/EZII (R^2^ < 0.01). Specifically, some eyes exhibited rapid overall GA growth accompanied by minimal MTII/EZII loss (“Rapid Grower, Slow Loser”), while others demonstrated slow overall GA growth but substantial MTII/EZII loss (“Slow Grower, Rapid Loser”). This discrepancy underscores the heterogeneous progression patterns in eyes with GA, suggesting that GA growth alone may not fully capture the dynamics of macular preservation.

**Figure 5:**
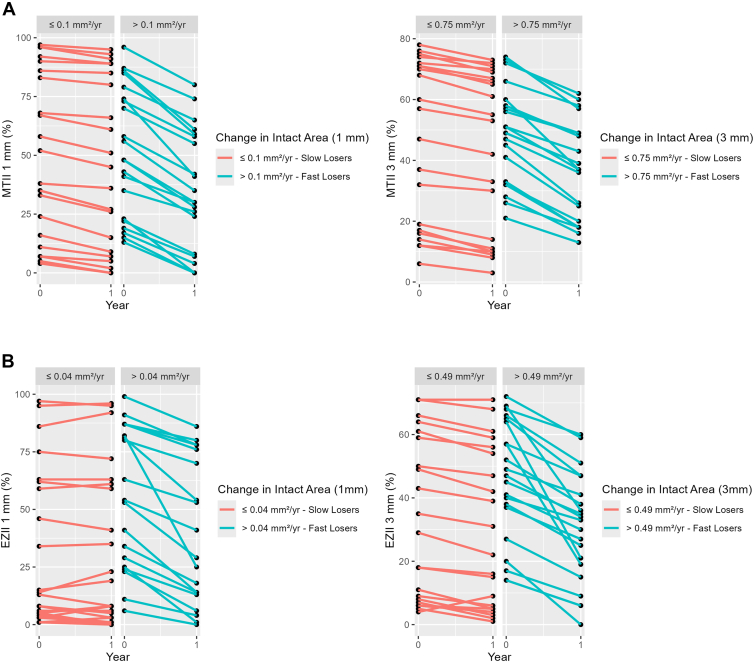
**A,** Baseline MTII values compared with loss of intact area over 1-year follow-up. **B,** Baseline EZII values compared with loss of intact EZ area over 1-year follow-up. EZ = ellipsoid zone; EZII = Ellipsoid Zone Integrity Index; MTII = Macular Tissue Integrity Index.

We further explored whether baseline MTII and EZII levels were predictive of subsequent macular tissue loss. Figure 5A stratifies participants based on their annual MTII decline (≤0.1 mm^2^/year and >0.1 mm^2^/year for the 1-mm circle and ≤0.75 mm^2^/year and >0.75 mm^2^/year for the 3-mm circle) and illustrates the change in intact macular tissue within the respective circles over 1 year. Figure 5B stratifies participants based on their annual EZII decline (≤0.04 mm^2^/year and >0.04 mm^2^/year for the 1-mm circle and ≤0.49 mm^2^/year and >0.49 mm^2^/year for the 3-mm circle) in a similar analysis. Regardless of the starting baseline MTII or EZII values, substantial variability in macular tissue loss was observed across participants. Some individuals with high baseline integrity still experienced significant loss, while others with lower baseline intact macula exhibited slower progression.

Change in MTII and EZII was not significantly associated with change in BCVA or LLVA at 1 year (R^2^ < 0.01–0.06). Across both 1-mm and 3-mm analyses, correlations were weak and not clinically meaningful. These findings suggest that macular tissue loss is independent of both baseline integrity and GA area growth, emphasizing the heterogeneous nature of disease progression and the complexities of structure–function correlations.

### Reproducibility

To assess the reproducibility of MTII and EZII grading methodologies, a subset was independently graded by both readers. Baseline FAF images showed a mean difference in intact area of 0.07 mm^2^ (SD 0.57) in the 3-mm circle and 0.00 mm^2^ (SD 0.55) in the 1-mm circle. Baseline OCT images showed a mean difference in intact area of 0.52 mm^2^ (SD 0.88) in the 3-mm circle and 0.08 mm^2^ (SD 0.14) in the 1-mm circle.

## Discussion

This introductory proof-of-concept study investigated the use of MTII and EZII as potential biomarkers of visual function. These metrics, measured within 1-mm and 3-mm rings around the fovea using FAF and OCT, capture similar features of macular health and demonstrate cross-sectional correlations with VA. Specifically, MTII and EZII within the central 1-mm correlate with BCVA, whereas the 3-mm measurements are associated with LLVA, highlighting their relevance to distinct visual functions. However, their longitudinal changes, including associations with VA decline and GA growth, are more nuanced.

Our data align with previous studies, showing that GA grows at an annual rate of approximately 2.0 mm^2^, and neither baseline GA area nor changes in GA area correlated with VA, underscoring the limitation of using GA area alone as a functional end point.^17^ Slowing the progression of GA toward the fovea may offer a more patient-centered and functionally meaningful outcome, preserving central vision and maintaining quality of life.^18^^,^^19^

The observed differences in correlations between BCVA and LLVA with the ring size may be attributed to the distribution of photoreceptors as shown by Curcio et al.^20^ Cones, concentrated in the central macula (1-mm circle), are critical for fine detail vision, and structural loss in this region is closely associated with BCVA decline. Conversely, rods, which are more prevalent in the parafoveal region (3-mm circle), primarily mediate night vision, explaining the stronger association between 3-mm metrics and LLVA. In fact, LLVA has been shown to have better correlation with fovea sparing GA.^21^ These findings highlight that MTII and EZII capture functionally relevant macular changes, with location-dependent associations.

Despite strong cross-sectional correlations, longitudinal associations between changes in MTII and VA were weaker. This may reflect the complex nature of GA progression and correlation with vision. Small peripheral MTII losses within the central 1-mm ring may not significantly impact BCVA if the fovea remains intact, and functional decline may lag behind structural damage.^22^^,^^23^ Additionally, the development of eccentric fixation and the need for larger sample sizes may influence these findings.^24^

To understand the profile of eyes with macular integrity decline, particularly those at risk for rapid structural loss, we evaluated 2 potential predictors: baseline intact area and baseline GA area. Both baseline MTII and EZII values did not predict subsequent macular tissue loss. Regardless of baseline tissue levels, some individuals lost tissue rapidly (“Rapid Losers”), while others experienced slower decline (“Slow Losers”). Previous studies using color photographs found similar associations between residual central macular tissue and foveal involvement.^25^^,^^26^

We also analyzed the relationship between changes in MTII/EZII and GA area growth over 1 year. Our findings demonstrate a lack of correlation between GA growth and MTII/EZII loss which can be explained by multiple studies that have shown that GA expands faster peripherally than centrally.^2^^,^^27^ Macular integrity appears to depend on the type of GA growth, which could be macula-sparing, macula-centric, or a mixed pattern of peripheral and central enlargement. Additionally, fragmented growth, where small multifocal lesions coalesce, or the de novo appearance of multifocal lesions within the central 1 mm disrupting macular metrics, adds further complexity. These variations illustrate that GA growth does not follow a straightforward narrative, and the relationship between GA growth and MTII/EZII is multi-faceted. The heterogeneous progression patterns of GA observed in this study may reflect underlying biological differences in lesion behavior. For example, rapid GA growth with minimal central loss (“Rapid Growers, Slow Losers”) could result from centrifugal spread that initially spares central photoreceptors, perhaps due to junctional zone activity or choroidal perfusion. In contrast, slow GA growth with substantial central tissue loss (“Slow Growers, Rapid Losers”) may involve early centripetal progression or central multifocal atrophy, potentially linked to subfoveal vulnerability.^13^^,^^20^^,^^28^ The presence of reticular pseudodrusen, differences in inflammation, or genetic predisposition may also influence these trajectories. These observations highlight the heterogeneity of GA progression, and that GA growth alone is insufficient to predict macular tissue loss.

The strength of this study is its multimodal approach to assessing macular status using both FAF and OCT. While EZII measured on OCT offers clearer insights into photoreceptor health, the biomarker is dependent on special software or artificial intelligence algorithms that are not yet widely available. In contrast, MTII measurement on FAF is more scalable and feasible for larger studies. There are also several limitations to consider. First, the relatively small sample size of this study precludes generalization to a larger population. Second, a 1-year follow-up period may fail to capture the full extent of GA progression or the long-term consequences GA has on visual function. Finally, functional tests such as contrast sensitivity and microperimetry were unavailable for participants in this study. As previous work has shown an association between GA area growth and a decline in visual function on both tests, the inclusion of functional tests may have revealed subtle visual acuity deficits not identified by standard testing methods used in this study.^29^^,^^30^

Macular Tissue Integrity Index and EZII represent promising conceptual biomarkers that warrant further validation in larger cohorts over longer periods of follow-up. Future studies should focus on key questions, such as understanding the natural history of macular tissue loss and identifying profiles of eyes prone to rapid decline in area of preserved macula, as enrolling these individuals in trials could provide critical insights. Additionally, understanding macular tissue loss patterns—concentric, sectoral, or asymmetric—may reveal important structural predictors of visual decline. Furthermore, the inclusion of emerging functional tests such as microperimetry would add additional insight into the structure-function relationship in macular degeneration. Despite over a decade of data on GA area enlargement and associated risk factors, no meaningful correlations with visual outcomes have been established. It is time to investigate these promising new metrics which have potential to redefine how we monitor and understand GA.

## Declaration of Generative AI and AI-Assisted Technologies in the Writing Process

During the preparation of this work the author(s) used ChatGPT for grammar and spelling check. After using this tool/service, the authors reviewed and edited the content as needed and take full responsibility for the content of the publication.
