# Supplementary material for: Investigating Macular Tissue Integrity Index as a Novel Biomarker in Geographic Atrophy
Source: Ophthalmol Sci. 2025 Jun 30;5(6):100871. doi: 10.1016/j.xops.2025.100871 (PMC12396552; doi:10.1016/j.xops.2025.100871)

Supplemental Figure 1: Relationship between low luminance visual acuity (LLVA) and structural imaging biomarkers. (A) LLVA versus macular tissue integrity index (MTII) within central 1mm and 3mm zones; (B) LLVA versus ellipsoid zone integrity index (EZII) within central 1mm and 3mm zones; (C) LLVA versus geographic atrophy (GA) area measured on fundus autofluorescence (FAF) and optical coherence tomography (OCT).

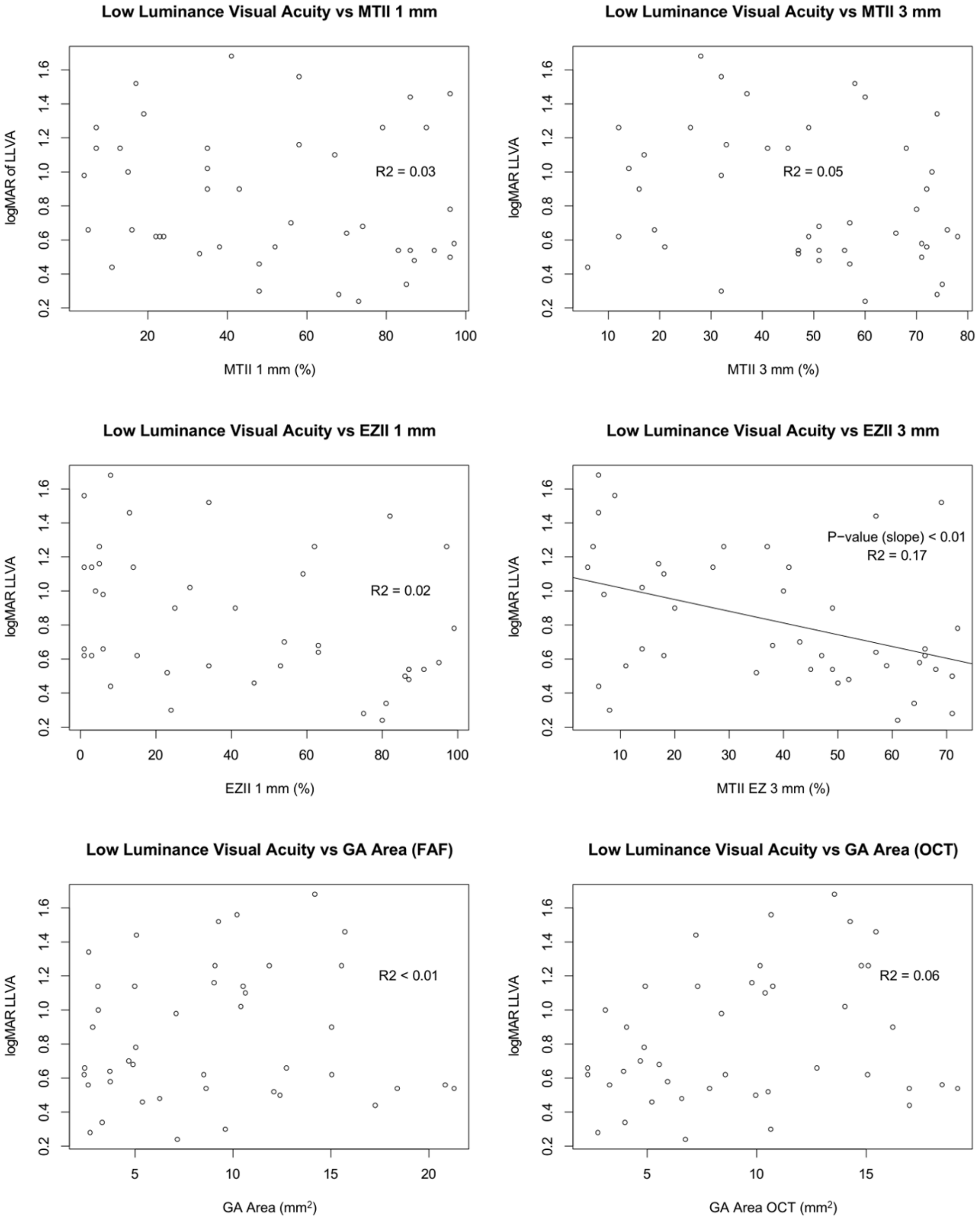

Supplement: Figure S1 [file mmc1.pdf]
